# DHA Induces Cell Death through the Production of ROS and the Upregulation of CHOP in Fibroblast-like Synovial Cells from Human Rheumatoid Arthritis Patients

**DOI:** 10.3390/ijms24021734

**Published:** 2023-01-15

**Authors:** Mini Jeong, Jong-Il Shin, Jaewook Cho, Yong-Joon Jeon, Jin-Hyun Kim, Jeehee Youn, Kyungho Lee

**Affiliations:** 1 Division of Allergy and Clinical Immunology, Department of Internal Medicine, Asan Medical Center, University of Ulsan College of Medicine, Seoul 05505, Republic of Korea; 2 Department of Biological Sciences, Konkuk University, 120 Neungdong-ro, Gwangin-gu, Seoul 05029, Republic of Korea; 3 Department of Anatomy and Cell Biology, College of Medicine, Hanyang University, Seoul 04763, Republic of Korea; 4 Korea Hemp Institute, Konkuk University, 120 Neungdong-ro, Gwangin-gu, Seoul 05029, Republic of Korea

**Keywords:** apoptosis, CHOP, DHA, ER stress, RA-FLSs, ROS

## Abstract

Rheumatoid arthritis (RA) is an inflammatory disease marked by a massive proliferation of synovial cells in the joints. In this study, we investigated the pro-apoptotic effects of docosahexaenoic acid (DHA) in human fibroblast-like synovial cells from RA patients (RA-FLS). An in vitro study using MH7A cells showed that DHA treatment induced caspase-8-dependent apoptosis in a dose-dependent manner and reduced the TNF-α-mediated induction of MMP-9 and IL-1β. DHA also induced the phosphorylation of eIF2α, the expression of the ER stress markers ATF4 and C/EBP homologous protein (CHOP), and death receptor 5 (DR5). The knockdown of CHOP or DR5 increased cell viability and reduced apoptosis in DHA-treated cells. Furthermore, the knockdown of CHOP reduced DHA-mediated DR5 expression, while the overexpression of CHOP increased DR5 expression. We also found that DHA treatment induced the accumulation of reactive oxygen species (ROS), and pretreatment with the anti-oxidant Tiron effectively abrogated not only the expression of CHOP and DR5, but also DHA-induced apoptosis. Under this condition, cell viability was increased, while PARP-1 cleavage and caspase-8 activation were reduced. All the findings were reproduced in human primary synovial cells obtained from RA patients. These results suggest that the DHA-mediated induction of ROS and CHOP induced apoptosis through the upregulation of DR5 in RA-FLSs, and that CHOP could be used as a therapy for RA.

## 1. Introduction

Rheumatoid arthritis (RA) is an inflammatory autoimmune disease characterized by chronic inflammation, hyperplasia of the synovial tissues, synovitis, the production of autoantibodies and cytokine, and irreversible destruction of the bone and cartilage of joints, which results in failure to move with severe pain [1,2,3,4]. The synovial hyperplasia is caused by synovial inflammation and the uncontrolled proliferation of the synovial fibroblasts in patients with rheumatoid arthritis (RA-FLS). This abnormal proliferation and invasion, even without specific growth factors, is characteristics of the RA-FLSs shared with cancer cells [5,6,7,8]. Thus, some anticancer drugs, such as paclitaxel (Taxol) and beta-elemene, have been shown to induce mitotic arrest and apoptosis in proliferating synoviocytes, but caused some side effects [9,10]. Many drugs, such as corticosteroids and nonsteroidal anti-inflammatory drugs (NSAIDs), that have been used to treat RA also caused toxicities including, the increased risk of heart attack and gastrointestinal damage [11,12,13].

Therefore, the search for agents to control abnormal proliferation and/or inflammation of RA-FLS with low or no toxicity is highly demanded. One example of this type of agents is omega-3 polyunsaturated fatty acids (PUFAs). Its function as a therapeutic drug for inflammation in neurodegenerative disorders, autoimmune diseases, or cancer has been described [14,15]. Previously, we have shown that hempseed oil, which contains high percentage of PUFAs, induces ROS- and C/EBP homologous protein (CHOP)-mediated apoptosis in MH7A RA-FLSs [16]. Two omega-3 FAs known to be used to improve RA symptoms are docosahexaenoic acid (DHA, C22:6n-3) and eicosapentaenoic acid (EPA, C20:5n-3). DHA is rich in fish oil and is converted from α-linolenic acid (ALA, 18:3n-3) in human cells by lipid metabolic enzymes [17]. The beneficial effects of omega-3 fatty acids are related to the altered expressions of genes encoding inflammatory mediators. For examples, DHA induces the downregulation of the vascular cell adhesion molecule (VCAM)-1 in endothelial cells [18]. EPA and DHA can inhibit the production of IL-1β and TNF-α by monocytes [19] and the production of IL-6 and IL-8 by endothelial cells [20,21].

Although DHA is known to increase apoptotic cell death in many cancer cells, such as colon cancer, prostate cancer, hepatocarcinoma, and lung carcinoma, as well as in normal cells, the mechanism of DHA in RA is not known. One cellular function of DHA is the induction of the unfolded protein response (UPR), which is known to be activated by the perturbation of homeostasis in the endoplasmic reticulum (ER). The mammalian UPR is initiated by the activation of the three sensor proteins, inositol-requiring enzyme 1α (IRE1α), activating transcription factor 6 (ATF6), and PKR-like ER kinase (PERK) [22,23]. The activated PERK phosphorylates α subunit of eukaryotic translation initiation factor 2 (eIF2α), leading to the attenuation of general protein synthesis, while allowing selective translation of particular mRNAs, such as activating transcription factor 4 (ATF4) mRNA [24]. One of the factors functioning downstream of ATF4 is CHOP, also known as growth arrest and DNA damage 153 (GADD153). It is a transcription factor that controls the expression of genes involved in cell survival or cell death [25,26]. CHOP functions as a proapoptotic factor by downregulating Bcl-2 expression through dimerization with C/EBPβ, by upregulating DR5, by disrupting the cellular redox state via the depletion of cellular glutathione, and by promoting mitochondrial cell death [25,27,28]. Despite evidence that CHOP acts as a proapoptotic factor, CHOP-mediated apoptosis is controversial because there is evidence that CHOP acts as an inflammatory regulator without inducing apoptosis in various types of inflammatory cells [29,30].

In this study, we investigated the molecular mechanism underlying the pro-apoptotic effects of DHA in MH7A synoviocytes (RA-FLSs) and primary synovial cells obtained from RA patients. We showed that DHA induced caspases-dependent apoptosis and ROS generation-mediated ER stress. In this situation, CHOP worked as a pro-apoptotic factor by mediating DHA-induced apoptosis.

## 2. Results

### 2.1. DHA Induces Caspase-Dependent Apoptosis and Reduces TNF-α-Mediated Inflammation in MH7A Cells

**Figure 1:**
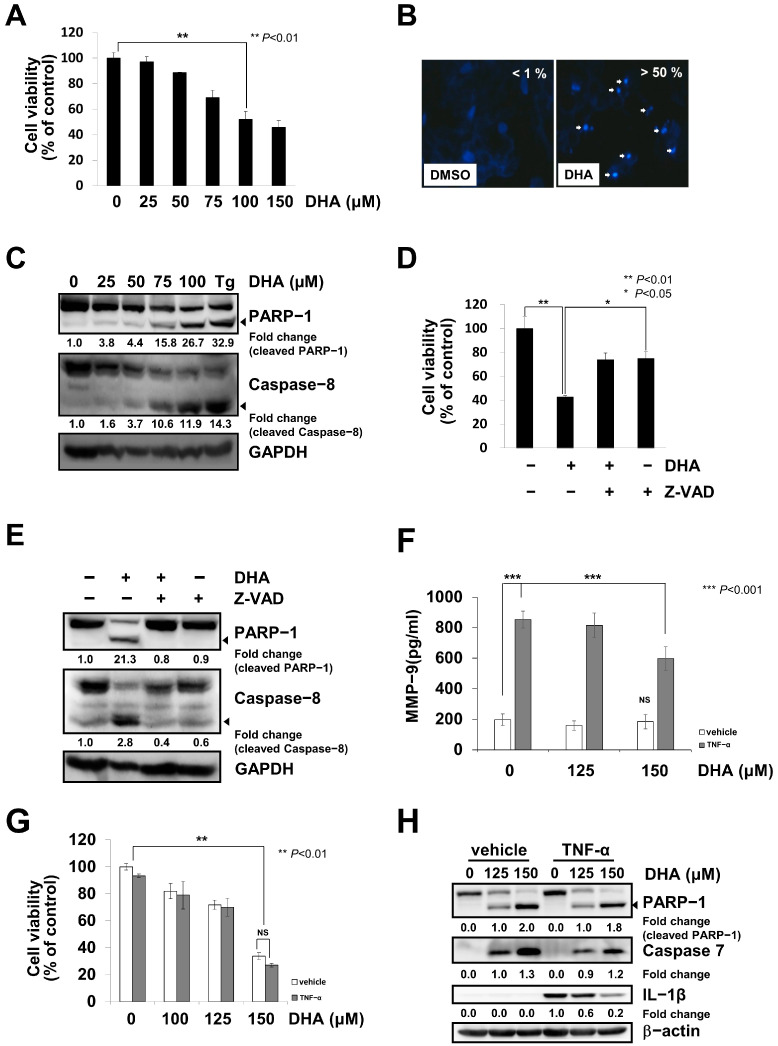
DHA induced caspase-dependent apoptosis and reduced TNF-α-mediated inflammation in MH7A cells. (**A**) Cells were treated with various concentrations of DHA for 24 h, and cell viability was determined using the Ez-cytox assay, as described in the Materials and Methods. Data represent the means ± S.D. of three independent experiments, performed in triplicate. A significant difference is indicated as ** *p* < 0.01. (**B**) Cells treated with 100 μM DHA, or DMSO as a control, for 24 h were fixed and stained with DAPI, and chromatin condensation was observed under the microscope (400×). Arrows indicate the condensed chromatins. (**C**) Cells were treated with various concentrations of DHA or 300 nM thapsigargin (Tg) for 24 h, as indicated, and cell lysates were subjected to SDS-PAGE, followed by immunoblot analysis using antibodies specific to PARP-1, caspase-8, or GAPDH. Arrows indicate the active form of the proteins. Blots have been cropped down to size for clarity. (**D**) Cells pre-treated with or without 5 μM Z-VAD-FMK (Z-VAD), a pan-caspase inhibitor, for 1 h were treated with 100 μM DHA for 24 h. Cell viability was determined using the Ez-cytox assay. Significant differences are indicated as * *p* < 0.05 or ** *p* < 0.01. (**E**) The cells obtained in (**D**) were lysed and subjected to SDS-PAGE, followed by immunoblot analysis using antibodies specific to PARP-1, caspase-8, or GAPDH. Arrows indicate the active form of the proteins. Blots have been cropped down to size for clarity. (**F**–**H**) Cells pre-treated with or without 10 ng/mL TNF-α for 30 min were treated with various concentrations of DHA for 24 h. (**F**) MMP-9 concentrations were measured using the sandwich ELISA assay kit. Significant differences are indicated as *** *p* < 0.001; NS, not significant. (**G**) Cell viability was determined using the MTT assay. Significant differences are indicated as ** *p* < 0.01. (**H**) Cell lysates were subjected to SDS-PAGE, followed by immunoblot analysis using antibodies specific to PARP-1, caspase-7, IL-1β, or GAPDH. Blots have been cropped down to size for clarity.

In this study, we investigated the pro-apoptotic effects of DHA, one of the omega-3 fatty acids, in RA-FLS MH7A cells and primary synovial cells. When the MH7A cells were treated with various concentrations of DHA for 24 h, cell viability was significantly reduced (Figure 1A). DAPI staining results showed that DHA treatment caused chromatin condensation in the MH7A cells (Figure 1B). DHA treatment also induced PARP-1 cleavage and caspase-8 activation in the MH7A cells (Figure 1C and Figure S1A). When MH7A cells pre-treated with a pan-caspase inhibitor Z-VAD-FMK for 1 h were treated with DHA for 24 h, cell viability was significantly recovered, and neither PARP-1 cleavage nor caspase-8 activation were detected (Figure 1D,E and Figure S1B). These results suggest that DHA-mediated cell death is caused by caspase-dependent apoptosis in MH7A cells. To test whether DHA-mediated effects are specific to RA-FLS, we investigated DHA-mediated apoptosis and cell viability using the A549 cell line. DHA treatment reduced the viability of A549 cells, during which PARP-1 cleavage and CHOP induction were observed (Figure S7). Therefore, DHA-induced apoptosis is not considered a cell-specific effect. To investigate whether DHA could affect the pathological activity induced by proinflammatory factors, we analyzed MMP-9, IL-1β production, cell viability, and caspase activation in cells treated with TNF-α and DHA. The induction of MMP-9 by TNF-α stimulation was reduced by approximately 30% with DHA treatment (Figure 1F). The DHA-mediated reduction of cell viability and caspase activation was not affected by TNF-α treatment (Figure 1G,H and Figure S1C). The TNF-α-mediated induction of IL-1β was significantly reduced by DHA treatment (Figure 1H and Figure S1C). These results suggest that DHA treatment could reduce cytokine-mediated inflammatory responses.

### 2.2. DHA Induced the Unfolded Protein Response (UPR) in MH7A Cells

**Figure 2:**
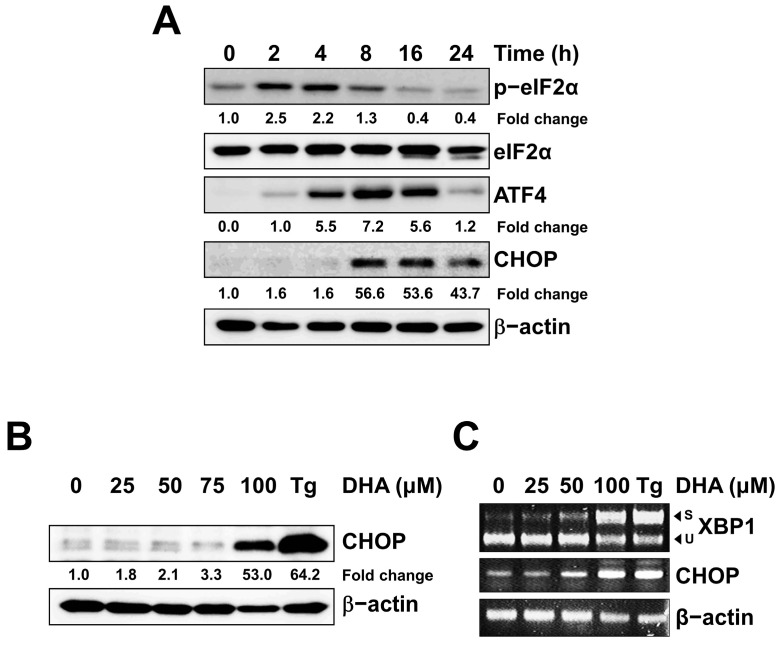
DHA induced the unfolded protein response in MH7A cells. Cells were treated with 130 μM DHA for various periods, as indicated (**A**), or with various concentrations of DHA or 300 nM Tg for 24 h (**B**) or 12 h (**C**). (**A**,**B**) Cell lysates were subjected to SDS-PAGE, followed by immunoblot analysis using antibodies specific to the phosphorylated form of eIF2α (p-eIF2α), eIF2α, ATF4, CHOP, or β-actin. Blots have been cropped down to size for clarity. (**C**) Total RNA was isolated, and RT-PCR analysis was performed using primers specific to XBP1, CHOP, or β-actin. The amplified XBP1 DNA fragments were digested with *Pst*I to distinguish the spliced (S) form and unspliced (U) form of XBP1 fragments, as indicated with arrows.

Since DHA has been reported to induce ER stress and cell death in some cells [31,32], we tested whether DHA treatment induces ER stress and the UPR in MH7A cells. Expression of the phosphorylated form of eIF2α, ATF4, and CHOP was increased by DHA treatment (Figure 2A and Figure S2A). Similarly, CHOP was increased by DHA treatment in a dose-dependent manner (Figure 2B and Figure S2B). The phosphorylation of eIF2α preceded the induction of ATF4 and CHOP, as reported previously (Figure 2A). When RT-PCR analysis was performed with total RNAs isolated from the MH7A cells, splicing of XBP1 mRNA and CHOP transcription was increased by DHA treatment (Figure 2C), indicating that DHA treatment induces the UPR response in MH7A cells.

### 2.3. CHOP Acted as a Pro-Apoptotic Factor in DHA-Treated MH7A Cells

**Figure 3:**
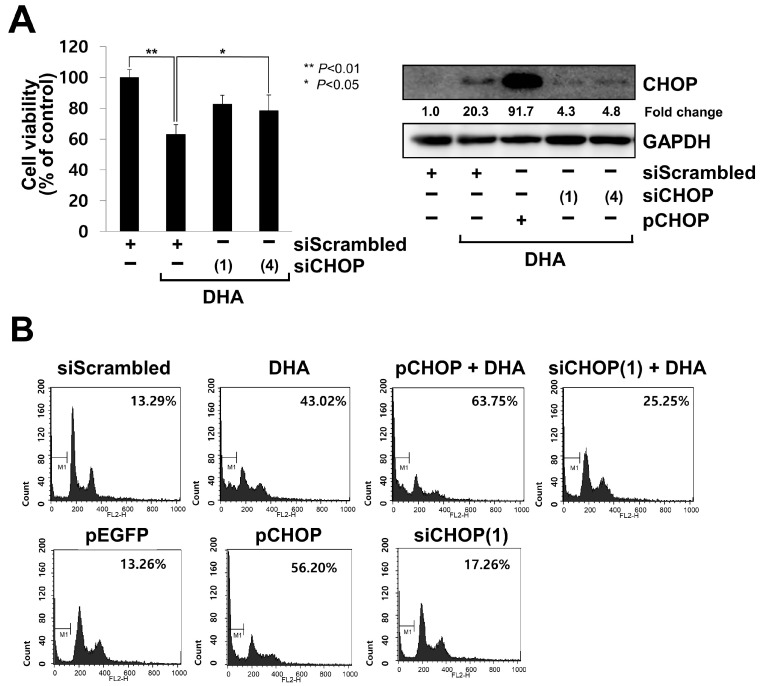
CHOP played a role as a pro-apoptotic factor in DHA-treated MH7A cells. The cells were transiently transfected with siRNA oligonucleotides specific for CHOP [siCHOP (1) or siCHOP(4)], scrambled oligonucleotides (siScrambled), a vector overexpressing CHOP (pCHOP), or EGFP (pEGFP). At 18 h post-transfection, the cells were treated with 130 μM DHA for 24 h. (**A**) Cell viability was determined using the Ez-cytox assay (**left**). Significant differences are indicated as * *p* < 0.05 or ** *p* < 0.01. Cell lysates were subjected to SDS-PAGE, followed by immunoblot analysis using antibodies specific to CHOP or GAPDH (**right**). Blots have been cropped down to size for clarity. (**B**) Cells were subjected to FACS analysis with PI staining. Apoptosis is expressed as the percentage of cells counted in the sub-G1 phase, as indicated by the bar in the histogram.

Since we observed the DHA-mediated induction of apoptosis and ER stress, we reasoned that ER stress resulted in the induction of apoptosis rather than the protection of the cells in our experimental conditions. Indeed, GRP78 is known as a pro-survival factor, while CHOP functions are viewed as a pro-apoptotic factor which acts by downregulating the transcription of Bcl-2 and by enhancing DR5 expression, leading to translocation of Bax from the cytosol to the mitochondria [25,26,28]. To test whether CHOP functions as a pro-apoptotic factor in DHA-treated MH7A cells, MH7A cells transfected with siRNA oligonucleotides specific for CHOP were treated with DHA for 24 h. The knockdown of CHOP significantly recovered cell viability under the DHA-treated condition (Figure 3A and Figure S3A). Consistent with this result, the overexpression of CHOP further increased the percentage of apoptotic cells, while the knockdown of CHOP decreased it in DHA-treated cells, as determined by the ratio of the number of sub-G1 cells to that of the whole cell population in the FACS analysis (Figure 3B, top). The overexpression of CHOP increased the apoptotic cell death, while the knockdown of CHOP did not significantly affect viability without DHA treatment, as noted in the FACS analysis (Figure 3B, bottom). These results suggest that CHOP acted as a pro-apoptotic factor in DHA-treated MH7A cells.

### 2.4. DR5 Is Involved in DHA-Induced Apoptosis in MH7A Cells

**Figure 4:**
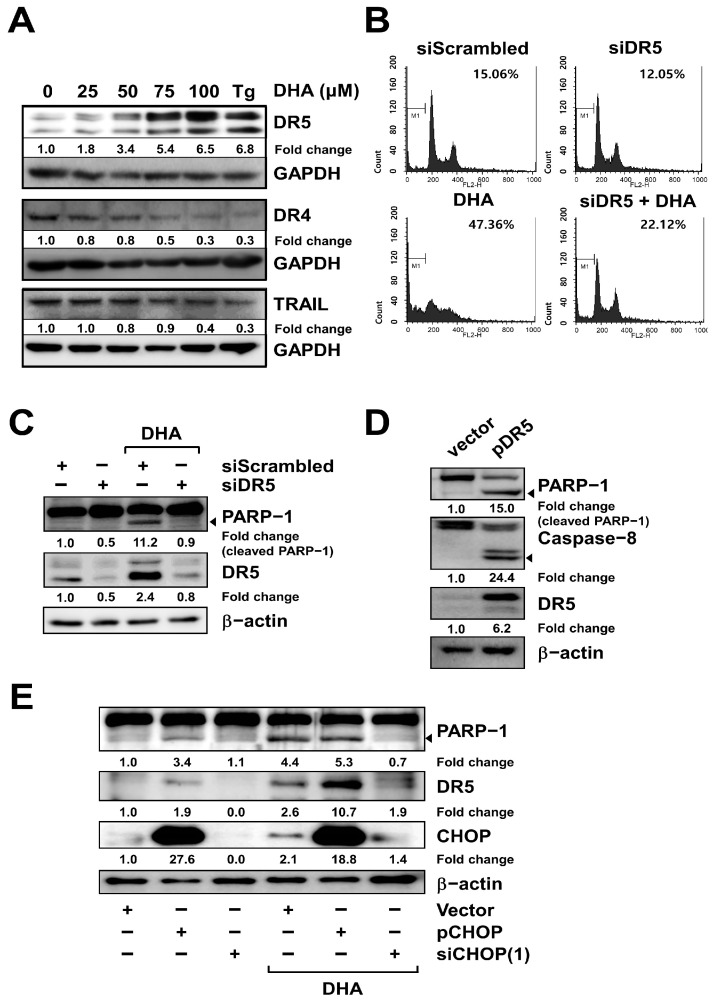
DR5 is involved in DHA-induced apoptosis in MH7A cells. (**A**) Cells were treated with various concentrations of DHA or 300 nM Tg for 24 h, as indicated. Cell lysates were subjected to SDS-PAGE, followed by immunoblot analysis using antibodies specific to DR5, DR4, TRAIL, or GAPDH. (**B**–**E**) Cells were transiently transfected with siRNA oligonucleotides specific for DR5 (siDR5), CHOP [siCHOP(1)], scrambled oligonucleotides (siScrambled), a plasmid overexpressing DR5 (pDR5), CHOP (pCHOP), or an empty pcDNA3.1 vector (Vector). At 18 h post-transfection, selected samples were treated with 130 μM DHA for 24 h, as indicated in B, C and E. (**B**) Cells stained with PI were subjected to FACS analysis. Apoptosis is expressed as the percentage of cells counted in the sub-G1 phase, as indicated by the bar in the histogram. (**C**–**E**) Cell lysates were subjected to SDS-PAGE, followed by immunoblot analysis using antibodies specific to PARP-1, DR5, or β-actin (**C**), PARP-1, caspase-8, DR5, or β-actin (**D**), PARP-1, DR5, CHOP, or β-actin (**E**). Arrows indicate the active form of the proteins. Blots have been cropped down to size for clarity.

The activation of caspase-8 is controlled by the extrinsic cell death pathway, which is composed of death receptors and ligand proteins such as DR5, DR4, and TRAIL [33]. Since we observed DHA-mediated caspase-8 activation in MH7A cells (Figure 1), as reported in other cell types [34,35], we tested to determine which proteins in the extrinsic death pathway function as pro-apoptotic factors mediating the effects of DHA in the MH7A cells. The expression of DR5 was increased by DHA treatment in a dose-dependent manner, while the expression of DR4 and TRAIL was decreased and unchanged, respectively (Figure 4A and Figure S4A). When DR5 knockdown cells were treated with DHA, the percentage of sub-G1 cells was decreased compared to the DHA-treated control cells in the FACS analysis, suggesting that the downregulation of DR5 reduced DHA-mediated apoptosis (Figure 4B, bottom). DR5 knockdown also reduced PARP-1 cleavage in DHA-treated cells (Figure 4C and Figure S4B, lanes 3 and 4). Considering that expression of TRAIL, a ligand of DR5, was detected, even without DHA treatment (Figure 4A), it would seem that binding of endogenously expressed TRAIL to DR5, which was induced by DHA treatment, induces apoptosis. Indeed, the overexpression of DR5 induced caspase-8 activation and apoptosis in MH7A cells (Figure 4D and Figure S4C). Since DR5 expression is reported to be controlled by CHOP, we checked the expression of DR5 under the condition that CHOP’s expression is modulated: the overexpression of CHOP increased DR5 expression, and the knockdown of CHOP decreased it, in the presence or absence of DHA treatment (Figure 4E and Figure S4D). DHA-induced PARP-1 cleavage was also decreased by the knockdown of CHOP (Figure 4E and Figure S4D). These results demonstrate that both the DHA-mediated induction of CHOP and the CHOP-mediated upregulation of DR5 are responsible for DHA-induced apoptosis in MH7A cells.

### 2.5. DHA-Mediated ROS Induced Apoptosis by Upregulation of CHOP and DR5 in MH7A Cells

**Figure 5:**
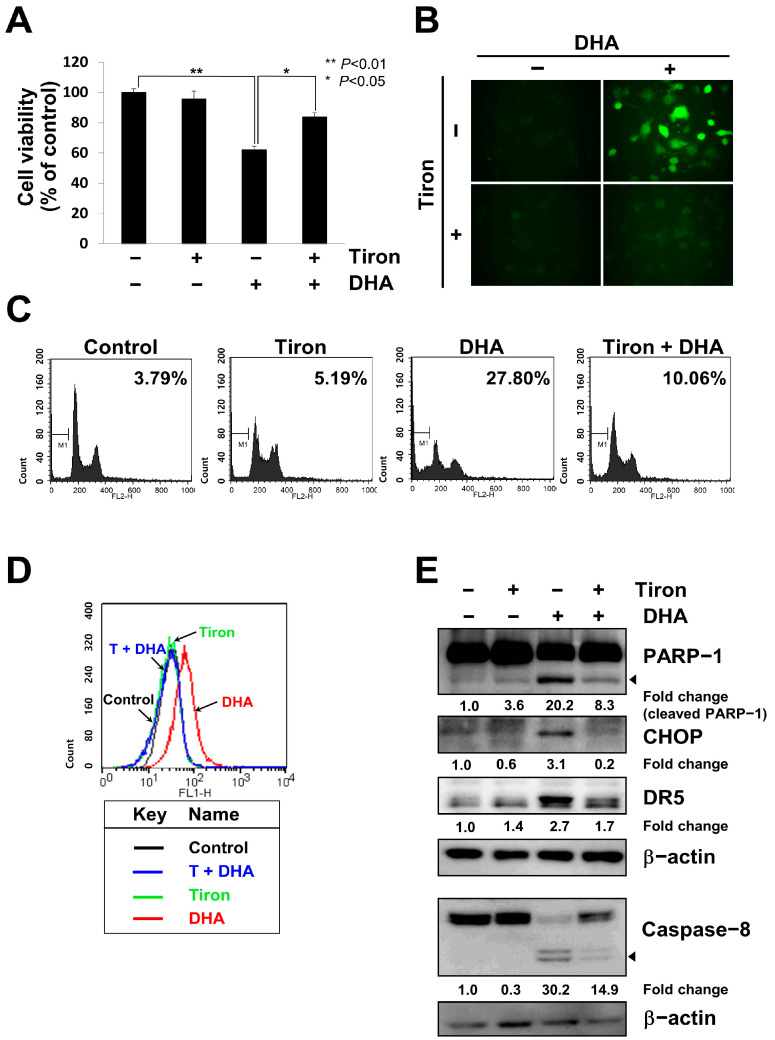
ROS induced apoptosis by increasing the expression of CHOP and DR5 in DHA-treated MH7A cells. (**A**) MH7A cells, pre-treated with or without 4 mM Tiron, an antioxidant, for 1 h were treated with 100 μM DHA for 24 h, and cell viability was determined using the Ez-cytox assay. (**B**) Cells were treated with 5 μM DCFH-DA for 30 min before treatment with Tiron, followed by treatment with 100 μM DHA for 2 h. Production of intracellular ROS was observed by fluorescence microscopy (400×). (**C**) Cells treated with 4 mM Tiron for 1 h and 130 μM DHA for 24 h were stained with PI and subjected to FACS analysis. Apoptosis is expressed as the percentage of cells counted in the sub-G1 phase, as indicated by the bar in the histogram. (**D**,**E**) MH7A cells pretreated with 5 μM DCFH-DA for 1 h were pretreated, with or without 4 mM Tiron, for 1 h, followed by treatment with 100 μM DHA for 2 h. The production of intracellular ROS was observed by FACS analysis (**D**). Cell lysates were subjected to SDS-PAGE, followed by immunoblot analysis using antibodies specific to PARP-1, CHOP, DR5, caspase-8, or β-actin (**E**). Arrows indicate the active form of the proteins. Blots have been cropped down to size for clarity.

Next, we tested ROS generation in DHA-treated MH7A cells because DHA is known to induce apoptosis and ROS in many types of cells [36]. When cells pre-treated with the antioxidant Tiron were treated with DHA, cell viability was significantly recovered (Figure 5A). In fluorescence microscopy using DCFH-DA, intercellular ROS was increased by DHA treatment, and pretreatment with Tiron prevented DHA-mediated ROS generation (Figure 5B). Treatment with Tiron also reduced DHA-mediated apoptotic cell death, as determined by the number of cells in the sub-G1 phase in FACS analysis using PI-stained cells (Figure 5C). FACS analysis also showed that DHA treatment induced intercellular ROS, which was abolished by Tiron treatment (Figure 5D). To test whether ROS generation causes CHOP induction and apoptosis, the cells treated with DHA, in the presence or absence of Tiron, were harvested and subjected to immunoblot analysis. All the DHA-mediated changes were prevented by Tiron treatment: PARP-1 cleavage was reduced, expression of CHOP and DR5 was reduced, and cleavage of caspase-8 was reduced (Figure 5E and Figure S5A). These results suggest that DHA induces the generation of ROS and it in turn induces the expression of CHOP and DR5, which work as pro-apoptotic factors in MH7A cells.

### 2.6. CHOP Induced Apoptosis in DHA-Treated Primary RA-FLSs Obtained from RA Patients

**Figure 6:**
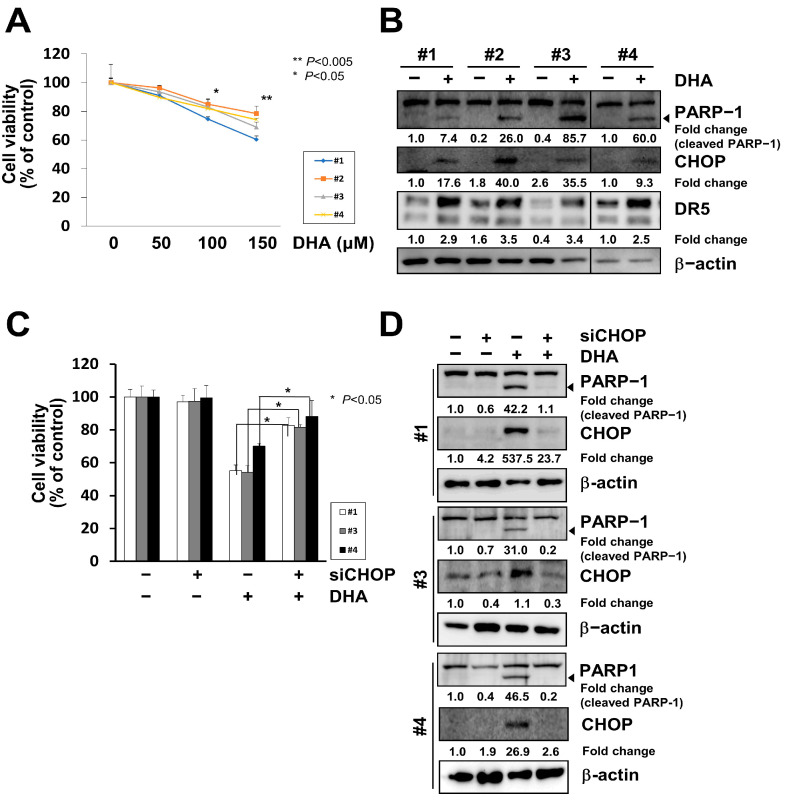
CHOP induced apoptosis in DHA-treated primary RA-FLSs obtained from RA patients. (**A**) Four lines of primary RA-FLS cells (#1–#4) were treated with various concentrations of DHA for 24 h, and cell viability was determined using the Ez-cytox assay. (**B**) Primary RA-FLS cells were treated with 150 μM DHA for 24 h, and cell lysates were subjected to SDS-PAGE, followed by immunoblot analysis using antibodies specific to PARP-1, CHOP, DR5, or β-actin. The arrow indicates the cleaved form of PARP-1. Blots have been cropped down to size for clarity. (**C**,**D**) Three lines of primary RA-FLS cells were transiently transfected with siRNA oligonucleotides specific for CHOP (siCHOP) or the scrambled oligonucleotides (siScrambled). At 18 h post-transfection, the cells were treated with 150 μM DHA for 24 h and cell viability was determined using the Ez-cytox assay (**C**). Cell lysates were subjected to SDS-PAGE, followed by immunoblot analysis using antibodies specific to PARP-1, CHOP, or β-actin (**D**). Arrows indicate the cleaved form of the PARP-1 proteins. Blots have been cropped down to size for clarity.

Lastly, we tested whether all the phenotypes observed in the MH7A cells could be reproduced in human primary synovial cells obtained from patients with RA. When four lines of primary RA-FLS cells were treated with various concentrations of DHA for 24 h, the viability of all four lines was reduced in a dose-dependent manner (Figure 6A). In immunoblot analysis performed with the lysates of DHA-treated cells, PARP-1 cleavage was increased, and the expression of CHOP, as well as DR5, was also increased (Figure 6B and Figure S6A). To verify the apoptotic roles of CHOP in primary RA-FLS cells, cells transiently transfected with siRNA oligonucleotides specific for CHOP (siCHOP) were treated with 150 μM DHA for 24 h. Cell viability was significantly recovered by CHOP knockdown in all cell lines (Figure 6C). Immunoblot assay results showed that PARP-1 cleavage was inhibited by the knockdown of CHOP in the DHA-treated cells (Figure 6D and Figure S6B), confirming that all the findings observed in the MH7A cells could be reproduced in human primary synovial cells obtained from patients with RA. These results suggest that CHOP and DR5 are important pro-apoptotic mediators working downstream of DHA in rheumatoid arthritis.

## 3. Discussion

In this study, during investigating the role of DHA on abnormal proliferation of synovial cells, we found that DHA treatment induces apoptosis in human synovial cells from patients with RA (RA-FLS, both in MH7A cells and primary RA-FLS) through induction of ROS, CHOP, and DR5, suggesting that CHOP functions as a pro-apoptotic factor.

Previously, the role of DHA in controlling abnormal proliferation has been reported in many types of cancer. DHA induces the dephosphorylation of GSK-3β, which results in the downregulation of β-catenin and TCF/LEF, and eventually, in the reduction in cell growth in hepatocellular carcinoma cells [37,38]. DHA also inhibits COX-2, an enzyme that converts free arachidonic acid (ARA) to PGE_2_. Since PGE_2_ upregulates β-catenin, the DHA-mediated inhibition of COX-2 results in the inhibition of cell growth. The DHA-mediated induction of 15-hydroxyprostaglandin dehydrogenase, which functions as an antagonist of COX-2, also contributes to cell growth reduction. In neuroblastoma cells, 17-HpDHA, one of the DHA derivatives, is known to induce cell cytotoxicity [39]. So far, it is not known whether β-catenin or 17-HpDHA function as anti-proliferative factors downstream of DHA in RA-FLS cells. Based on the fact that RA-FLS cells share some phenotypes with cancer cells, and that DHA induces apoptosis in RA-FLS cells (Figure 1, Figure 3, Figure 4, Figure 5 and Figure 6), it is likely that β-catenin or 17-HpDHA function similarly in both RA-FLS and cancer cells downstream of DHA.

One of the most important factors inducing synovial cell apoptosis would be DHA-induced ROS. The induction of ROS by DHA can be explained in several different ways. First, the accumulated DHA and PUFAs in the cell can be converted to peroxides and aldehyde products by the lipid peroxidation process. Second, DHA induces mitochondrial ROS generation and apoptosis in mutant p53-containing cancer cells [40]. Third, DHA is involved in intracellular ROS production via modulating NOX4 anion superoxide production or NADPH oxidase [41,42]. Finally, cell surface receptors for DHA, such as GPR120 and GPR40, could play roles in ROS production [43,44]. Although it is not clear which signal cascade makes the greatest contribution to the production of ROS under DHA-treated conditions, ROS must be an important factor mediating DHA-induced apoptosis because blocking ROS production by Tiron treatment efficiently abrogated apoptosis in DHA-treated cells (Figure 5).

Our results are inconsistent with the general consensus that RA treatment strategies should be directed towards the reduction of ROS, as the imbalanced oxidants/antioxidants ratio itself is elevated by a hypoxic in vivo environment of the inflamed joint. For example, it has been reported that the expression of peroxynitrite was increased via the induction of iNOS and NADPH oxidase (NOX) in hypoxic conditions [45], and the hypoxia-mediated induction of NOX-2 increased angiogenesis in the joints with inflammatory arthritis [46]. Moreover, ROS induced the expression of Cox-2 and prostaglandin E2 (PGE2) in bovine synovial fibroblast [47], and the inhibition of ROS using thymoquinone prevented RANKL-induced osteolysis by suppressing NF-κB and MAPK signaling in macrophages [48]. Considering the many reports stating that elevated ROS induced apoptosis in RA-FLS [10,16,49], the DHA-mediated induction of ROS and the resultant synovial cell apoptosis, as shown in this study, could be one way to reduce the symptoms of RA. Since our results were obtained by using cultured synovial cells, the DHA-mediated induction of ROS and apoptosis in vivo needs to be investigated.

The UPR is known to be activated by ROS, wherein ROS increases the phosphorylation of eIF2α, and the resultant upregulation of transcription factor ATF4 upregulates CHOP. From the results in this study, it could easily be inferred that DHA-induced ROS caused the upregulation of CHOP. One interesting finding concerns the apoptotic role of CHOP in RA-FLS. Although there are some debates regarding its role in apoptosis, CHOP seems to function as a proapoptotic factor that regulates the expression of genes involved in pro- and anti-apoptotic pathways. CHOP upregulates or downregulates many genes, including DR5, Bcl-2, and tRNA synthetase [50,51]. The CHOP-mediated upregulation of tRNA synthetase increases ROS production through oxidative protein folding in the ER [50]. CHOP itself can also be upregulated by endogenous ROS [52]. In this study, the induction of CHOP enhanced apoptosis, and its knockdown reduced apoptosis in DHA-treated RA-FLS (Figure 4 and Figure 6). Since the knockdown of CHOP did not rescue DHA-mediated phenotypes dramatically in terms of viability and the percentage of sub-G1 cells, despite the quite efficient inhibition of CHOP expression, CHOP might not be the major factor that mediates the apoptotic effects of DHA. Nevertheless, our results support that CHOP functions as a pro-apoptotic factor in DHA-treated RA-FLS cells.

The apoptotic role of CHOP seems to be mediated by DR5 induction in our experimental conditions (Figure 3E, Figure 4, Figure 5E and Figure 6B). DHA treatment induced the expression of CHOP and DR5, and the overexpression of CHOP induced the expression of DR5 (Figure 3E and Figure 4A). The overexpression of DR5 induced PARP-1 cleavage, and the knockdown of DR5 prevented PARP-1 cleavage and rescued DHA-mediated apoptosis (Figure 4B–D). Since the basal level expression of TARIL is clearly detected in the MH7A cells, we hypothesize that the interaction between endogenous TRAIL and upregulated DR5 induces apoptosis through the extrinsic death pathway in DHA-treated RA-FLS cells, as confirmed by the processing of caspase-8 (Figure 1C,E and Figure 5E). We cannot rule out the possibility that other death signaling cascades working through interaction of DHA with its receptors, such as GPR120 and GPR40, function to induce apoptosis.

In fact, the binding of PUFAs such as DHA to PPARγ, GPR40, and GPR120 inhibits inflammation. DHA-bound PPARγ forms a heterodimer with the retinoid X receptor (RXR) and inhibits inflammation the through transcriptional induction of genes involved in anti-inflammation, the reduction of NFκB translocation into nucleus, and the downregulation of TNF-α, IL-6, etc. Similarly, DHA-activated GPR120 inhibits the phosphorylation of IκB, which inhibits NFκB and the resultant reduction of cytokines, COX-2, iNOS, and MMP. Therefore, DHA has been used as a dietary supplement to improve the clinical effectiveness of RA treatment. It is interesting to note that DHA-treated RA-FLS cells showed the induction of CHOP and apoptosis. These results suggest that CHOP could serve as a therapeutic gene target of RA, and that the efficacy of DHA would be the most significant when applied to cells that show both abnormal proliferation and pro-inflammatory function, such as RA-FLS cells.

## 4. Materials and Methods

### 4.1. Chemicals and Reagents

DHA, DAPI, 4,5-dihydroxy-1,3-benzenedisulfonic acid (Tiron) and Oil red O were purchase from Sigma (St. Louis, MO, USA). The 2, 7-dichlorofluorescein diacetate (DCFH-DA) was purchased from Molecular Probes (Eugene, OR), and z-VAD-FMK was obtained from Calbiochem (La Jolla, CA, USA). The anti-caspase-8 antibody (9746) and the anti-CHOP/GADD153 (2859) antibodies were obtained from Cell signaling technology (Beverly, MA, USA). The anti-PARP-1 (sc-7150) was obtained from Santa Cruz Biotechnology (Santa Cruz, CA, USA). The anti-KDEL antibody (ADI-SPA-827) was obtained from Enzo Life Sciences (Farmingdale, NY, USA). The anti-DR5 antibody (AB16942) was purchase from Millipore (Burlington, MA, USA).

### 4.2. Cell Lines and Cell Culture

Human primary RA-FLSs were isolated from 4 patients with RA during joint surgery in the Department of Orthopedic Surgery at Hanyang University Hospital after written informed consent was obtained from all the patients. The experimental protocols were approved by the Institutional Review Board (IRB) of the Hanyang University Medical School, Korea (IRB permit #: HYI-13-097-1). All methods were carried out in accordance with approved guidelines and regulations. This manuscript does not contain any information that could lead to the identification of the participants. MH7A cells [53] and human primary RA-FLSs were grown in RPMI 1640 medium (WelGENE, Daegu, Republic of Korea) containing 10% heat-inactivated fetal bovine serum (GIBCO) and 1% penicillin/streptomycin mixtures (GIBCO) under a humidified atmosphere in 5% CO_2_ at 37 °C.

### 4.3. Measurement of Cell Viability

EZ-cytox (Daeiltech Co., Seoul, Republic of Korea) was used to determine the relative number of viable cells. MH7A cells were seeded into 48-well cell culture plates and incubated for 16 h. Cells were treated with various concentrations of DHA for 24 h. When needed, cells were pre-treated with the antioxidant Tiron for 1 h or transfected with siRNA oligos and plasmid for 18 h before DHA treatment. After washing the cells once with PBS, 300 μL of fresh medium was added to each well, along with 15 μL of EZ-cytox, and the mixture was incubated for 30 min at 37 °C. The absorbance of the soluble substrate was measured at 450/690 nm using an ELISA Reader (UYM 340; ASYS Hitech, Salzburg, Austria).

### 4.4. Plasmids, siRNA Oligos, and Transfection

RNAs were isolated from thapsigargin-treated MH7A cells, and CHOP cDNA was obtained by RT-PCR. A 0.5-kb *BamH*I–*Hind*III fragment was subcloned into the pcDNA 3.1 plasmid, as described [16]. siRNA oligonucleotides specific for CHOP [siCHOP(1) and siCHOP(4)], scrambled oligonucleotides (siScrambled), and the transfection of the plasmids were described previously [16].

### 4.5. Microscopic Determination of Apoptosis and Immunoblot Analysis

The identification of apoptotic cells by fluorescence microscopy and immunoblot analysis were described previously [16]. The protein bands were quantified using Image Quant TL software (Cytiva).

### 4.6. Fluorescence-Activated Cell Sorting (FACS) Analysis

Attached and floating cells grown in 60 mm culture dishes were pooled in conical tubes, pelleted by centrifugation, washed in PBS, and fixed with cold 70% ethanol at 4 °C overnight. The cells were washed, resuspended in 1 mL of propidium iodide (PI) solution containing 20 μg/mL RNase A and 100 μg/mL PI, and incubated for 30 min at 37 °C. Apoptotic cells were assayed using a Becton Dickinson Flow Cytometer (Becton-Dickinson Biosciences, San Jose, CA, USA) at 488 nm, and data were analyzed with WinMDI version 2.9 Software (Joe Trotter, Scripps Research Institute, La Jolla, CA, USA). Sub-G1 cells were considered as apoptotic cells. The percentage of apoptotic cells was calculated as the ratio of the number of sub-G1 cells to that of the whole cell population.

### 4.7. Observation of Intracellular ROS Levels

Intracellular ROS production was detected using DCFH-DA as an intracellular fluorescence probe. Briefly, cells were treated with 5 μM DCFH-DA for 1 h at 37 °C. After washing twice with PBS, DHA was added to the medium for 3 h at 37 °C. When needed, Tiron was added 1 h before DHA treatment. The fluorescence intensity of DCF was observed under a fluorescence microscope (ECLIPSE TS-200; Nikon) using 488 nm for excitation.

### 4.8. Enzyme-Linked Immunosorbent Assay

MH7A cells, pre-treated with or without 10 ng/mL TNF-α for 30 min, were treated with various concentrations of DHA for 24 h. The cell culture medium was collected, and the level of MMP-9 was measured using a commercially available ELISA kit for MMP-9 (RayBiotech, Norcross, GA, USA), according to the manufacturer’s instructions.

### 4.9. Statistical Analysis

A one-way analysis of variance was used for all statistical analyses, with independent experiments. Following this, post hoc analysis (t-test or Duncan’s) was conducted to determine the minimum effective dose of DHA. *p* values that were less than 0.05 were considered to be significant, unless otherwise specified. All data were expressed as the mean ± S.D. of three independent experiments, performed in triplicate.

## 5. Conclusions

- DHA induced caspase-dependent apoptosis and reduces TNF-α-mediated inflammation in MH7A cells.
- DHA induced the unfolded protein response (UPR) in MH7A cells.
- CHOP acted as a pro-apoptotic factor in DHA-treated MH7A cells.
- DR5 was involved in DHA-induced apoptosis in MH7A cells.
- DHA-mediated ROS induced apoptosis by the upregulation of CHOP and DR5 in MH7A cells.
- CHOP induced apoptosis in DHA-treated primary RA-FLSs obtained from RA patients.

## Data Availability

The data presented in this study are available on request from the corresponding author.

## Supplementary Materials

The following supporting information can be downloaded at: https://www.mdpi.com/article/10.3390/ijms24021734/s1.

## References

[1] Choy E. (2012). Understanding the dynamics: Pathways involved in the pathogenesis of rheumatoid arthritis. Rheumatology.

[2] Scott D.L., Steer S. (2007). The course of established rheumatoid arthritis. Best Pract. Res. Clin. Rheumatol..

[3] Malaviya A.P., Ostor A.J. (2012). Rheumatoid arthritis and the era of biologic therapy. Inflammopharmacology.

[4] Liu H., Pope R.M. (2003). The role of apoptosis in rheumatoid arthritis. Curr. Opin. Pharmacol..

[5] Bottini N., Firestein G.S. (2013). Duality of fibroblast-like synoviocytes in RA: Passive responders and imprinted aggressors. Nat. Rev. Rheumatol..

[6] Baier A., Meineckel I., Gay S., Pap T. (2003). Apoptosis in rheumatoid arthritis. Curr. Opin. Rheumatol..

[7] Yamanishi Y., Firestein G.S. (2001). Pathogenesis of rheumatoid arthritis: The role of synoviocytes. Rheum. Dis. Clin. N. Am..

[8] Muller-Ladner U., Kriegsmann J., Franklin B.N., Matsumoto S., Geiler T., Gay R.E., Gay S. (1996). Synovial fibroblasts of patients with rheumatoid arthritis attach to and invade normal human cartilage when engrafted into SCID mice. Am. J. Pathol..

[9] Hui A., Kulkarni G.V., Hunter W.L., McCulloch C.A., Cruz T.F. (1997). Paclitaxel selectively induces mitotic arrest and apoptosis in proliferating bovine synoviocytes. Arthritis Rheum..

[10] Zou S., Wang C., Cui Z., Guo P., Meng Q., Shi X., Gao Y., Yang G., Han Z. (2016). beta-Elemene induces apoptosis of human rheumatoid arthritis fibroblast-like synoviocytes via reactive oxygen species-dependent activation of p38 mitogen-activated protein kinase. Pharmacol. Rep..

[11] Aygun D., Kaplan S., Odaci E., Onger M.E., Altunkaynak M.E. (2012). Toxicity of non-steroidal anti-inflammatory drugs: A review of melatonin and diclofenac sodium association. Histol. Histopathol..

[12] Lanas A. (2010). A review of the gastrointestinal safety data—A gastroenterologist’s perspective. Rheumatology.

[13] Bourre-Tessier J., Haraoui B. (2010). Methotrexate drug interactions in the treatment of rheumatoid arthritis: A systematic review. J. Rheumatol..

[14] Ruggiero C., Lattanzio F., Lauretani F., Gasperini B., Andres-Lacueva C., Cherubini A. (2009). Omega-3 polyunsaturated fatty acids and immune-mediated diseases: Inflammatory bowel disease and rheumatoid arthritis. Curr. Pharm. Des..

[15] Simopoulos A.P. (2002). Omega-3 fatty acids in inflammation and autoimmune diseases. J. Am. Coll. Nutr..

[16] Jeong M., Cho J., Shin J.I., Jeon Y.J., Kim J.H., Lee S.J., Kim E.S., Lee K. (2014). Hempseed oil induces reactive oxygen species- and C/EBP homologous protein-mediated apoptosis in MH7A human rheumatoid arthritis fibroblast-like synovial cells. J. Ethnopharmacol..

[17] Brenna J.T., Salem N., Sinclair A.J., Cunnane S.C. (2009). alpha-Linolenic acid supplementation and conversion to n-3 long-chain polyunsaturated fatty acids in humans. Prostaglandins Leukot Essent Fat. Acids.

[18] De Caterina R., Cybulsky M.I., Clinton S.K., Gimbrone M.A., Libby P. (1994). The omega-3 fatty acid docosahexaenoate reduces cytokine-induced expression of proatherogenic and proinflammatory proteins in human endothelial cells. Arterioscler. Thromb..

[19] Babcock T.A., Novak T., Ong E., Jho D.H., Helton W.S., Espat N.J. (2002). Modulation of lipopolysaccharide-stimulated macrophage tumor necrosis factor-alpha production by omega-3 fatty acid is associated with differential cyclooxygenase-2 protein expression and is independent of interleukin-10. J. Surg. Res..

[20] Novak T.E., Babcock T.A., Jho D.H., Helton W.S., Espat N.J. (2003). NF-kappa B inhibition by omega-3 fatty acids modulates LPS-stimulated macrophage TNF-alpha transcription. Am. J. Physiol. Lung Cell Mol. Physiol..

[21] Babcock T.A., Helton W.S., Hong D., Espat N.J. (2002). Omega-3 fatty acid lipid emulsion reduces LPS-stimulated macrophage TNF-alpha production. Surg. Infect..

[22] Kaufman R.J. (1999). Stress signaling from the lumen of the endoplasmic reticulum: Coordination of gene transcriptional and translational controls. Genes Dev..

[23] Kaufman R.J., Scheuner D., Schroder M., Shen X., Lee K., Liu C.Y., Arnold S.M. (2002). The unfolded protein response in nutrient sensing and differentiation. Nat. Rev. Mol. Cell Biol..

[24] Wek R.C., Jiang H.Y., Anthony T.G. (2006). Coping with stress: eIF2 kinases and translational control. Biochem. Soc. Trans..

[25] Oyadomari S., Mori M. (2004). Roles of CHOP/GADD153 in endoplasmic reticulum stress. Cell Death Differ..

[26] Yamaguchi H., Wang H.G. (2004). CHOP is involved in endoplasmic reticulum stress-induced apoptosis by enhancing DR5 expression in human carcinoma cells. J. Biol. Chem..

[27] Kim R., Emi M., Tanabe K., Murakami S. (2006). Role of the unfolded protein response in cell death. Apoptosis Int. J. Program. Cell Death.

[28] McCullough K.D., Martindale J.L., Klotz L.O., Aw T.Y., Holbrook N.J. (2001). Gadd153 sensitizes cells to endoplasmic reticulum stress by down-regulating Bcl2 and perturbing the cellular redox state. Mol. Cell. Biol..

[29] Endo M., Mori M., Akira S., Gotoh T. (2006). C/EBP homologous protein (CHOP) is crucial for the induction of caspase-11 and the pathogenesis of lipopolysaccharide-induced inflammation. J. Immunol..

[30] Jeong M., Cho J., Cho W.-S., Shin G.-C., Lee K. (2009). The glucosamine-mediated induction of CHOP reduces the expression of inflammatory cytokines by modulating JNK and NF-κB in LPS-stimulated RAW264.7 cells. Genes Genom..

[31] Jakobsen C.H., Storvold G.L., Bremseth H., Follestad T., Sand K., Mack M., Olsen K.S., Lundemo A.G., Iversen J.G., Krokan H.E. (2008). DHA induces ER stress and growth arrest in human colon cancer cells: Associations with cholesterol and calcium homeostasis. J. Lipid Res..

[32] Prasad A., Bloom M.S., Carpenter D.O. (2010). Role of calcium and ROS in cell death induced by polyunsaturated fatty acids in murine thymocytes. J. Cell Physiol..

[33] O’Flaherty J., Mei Y., Freer M., Weyman C.M. (2006). Signaling through the TRAIL receptor DR5/FADD pathway plays a role in the apoptosis associated with skeletal myoblast differentiation. Apoptosis.

[34] Fukui M., Kang K.S., Okada K., Zhu B.T. (2013). EPA, an omega-3 fatty acid, induces apoptosis in human pancreatic cancer cells: Role of ROS accumulation, caspase-8 activation, and autophagy induction. J. Cell Biochem..

[35] Xiong A., Yu W., Tiwary R., Sanders B.G., Kline K. (2012). Distinct roles of different forms of vitamin E in DHA-induced apoptosis in triple-negative breast cancer cells. Mol. Nutr. Food Res..

[36] Kang K.S., Wang P., Yamabe N., Fukui M., Jay T., Zhu B.T. (2010). Docosahexaenoic acid induces apoptosis in MCF-7 cells in vitro and in vivo via reactive oxygen species formation and caspase 8 activation. PLoS ONE.

[37] Lim K., Han C., Dai Y., Shen M., Wu T. (2009). Omega-3 polyunsaturated fatty acids inhibit hepatocellular carcinoma cell growth through blocking beta-catenin and cyclooxygenase-2. Mol. Cancer Ther..

[38] Lim K., Han C., Xu L., Isse K., Demetris A.J., Wu T. (2008). Cyclooxygenase-2-derived prostaglandin E2 activates beta-catenin in human cholangiocarcinoma cells: Evidence for inhibition of these signaling pathways by omega 3 polyunsaturated fatty acids. Cancer Res..

[39] Gleissman H., Yang R., Martinod K., Lindskog M., Serhan C.N., Johnsen J.I., Kogner P. (2010). Docosahexaenoic acid metabolome in neural tumors: Identification of cytotoxic intermediates. FASEB J..

[40] Shin S., Jing K., Jeong S., Kim N., Song K.S., Heo J.Y., Park J.H., Seo K.S., Han J., Park J.I. (2013). The omega-3 polyunsaturated fatty acid DHA induces simultaneous apoptosis and autophagy via mitochondrial ROS-mediated Akt-mTOR signaling in prostate cancer cells expressing mutant p53. Biomed. Res. Int..

[41] Wu H., Ichikawa S., Tani C., Zhu B., Tada M., Shimoishi Y., Murata Y., Nakamura Y. (2009). Docosahexaenoic acid induces ERK1/2 activation and neuritogenesis via intracellular reactive oxygen species production in human neuroblastoma SH-SY5Y cells. Biochim. Biophys. Acta.

[42] Rossary A., Arab K., Steghens J.P. (2007). Polyunsaturated fatty acids modulate NOX 4 anion superoxide production in human fibroblasts. Biochem. J..

[43] Shah B.P., Liu P., Yu T., Hansen D.R., Gilbertson T.A. (2012). TRPM5 is critical for linoleic acid-induced CCK secretion from the enteroendocrine cell line, STC-1. Am. J. Physiol. Cell Physiol..

[44] Oh D.Y., Talukdar S., Bae E.J., Imamura T., Morinaga H., Fan W., Li P., Lu W.J., Watkins S.M., Olefsky J.M. (2010). GPR120 is an omega-3 fatty acid receptor mediating potent anti-inflammatory and insulin-sensitizing effects. Cell.

[45] Chenevier-Gobeaux C., Simonneau C., Lemarechal H., Bonnefont-Rousselot D., Poiraudeau S., Rannou F., Anract P., Borderie D. (2012). Hypoxia induces nitric oxide synthase in rheumatoid synoviocytes: Consequences on NADPH oxidase regulation. Free Radic. Res..

[46] Biniecka M., Connolly M., Gao W., Ng C.T., Balogh E., Gogarty M., Santos L., Murphy E., Brayden D., Veale D.J. (2014). Redox-mediated angiogenesis in the hypoxic joint of inflammatory arthritis. Arthritis. Rheumatol..

[47] Onodera Y., Teramura T., Takehara T., Shigi K., Fukuda K. (2015). Reactive oxygen species induce Cox-2 expression via TAK1 activation in synovial fibroblast cells. FEBS Open Bio.

[48] Thummuri D., Jeengar M.K., Shrivastava S., Nemani H., Ramavat R.N., Chaudhari P., Naidu V.G. (2015). Thymoquinone prevents RANKL-induced osteoclastogenesis activation and osteolysis in an in vivo model of inflammation by suppressing NF-KB and MAPK Signalling. Pharm. Res..

[49] Yan C., Kong D., Ge D., Zhang Y., Zhang X., Su C., Cao X. (2015). Mitomycin C induces apoptosis in rheumatoid arthritis fibroblast-like synoviocytes via a mitochondrial-mediated pathway. Cell. Physiol. Biochem. Int. J. Exp. Cell. Physiol. Biochem. Pharmacol..

[50] Han J., Back S.H., Hur J., Lin Y.H., Gildersleeve R., Shan J., Yuan C.L., Krokowski D., Wang S., Hatzoglou M. (2013). ER-stress-induced transcriptional regulation increases protein synthesis leading to cell death. Nat. Cell Biol..

[51] Malhi H., Kaufman R.J. (2011). Endoplasmic reticulum stress in liver disease. J. Hepatol..

[52] Thevenot P.T., Sierra R.A., Raber P.L., Al-Khami A.A., Trillo-Tinoco J., Zarreii P., Ochoa A.C., Cui Y., Del Valle L., Rodriguez P.C. (2014). The stress-response sensor chop regulates the function and accumulation of myeloid-derived suppressor cells in tumors. Immunity.

[53] Miyazawa K., Mori A., Okudaira H. (1998). Establishment and characterization of a novel human rheumatoid fibroblast-like synoviocyte line, MH7A, immortalized with SV40 T antigen. J. Biochem.

