# Supplementary material for: DHA Induces Cell Death through the Production of ROS and the Upregulation of CHOP in Fibroblast-like Synovial Cells from Human Rheumatoid Arthritis Patients
Source: Int J Mol Sci. 2023 Jan 15;24(2):1734. doi: 10.3390/ijms24021734 (PMC9865349; doi:10.3390/ijms24021734)
Supplement: Supplementary file 1 [file ijms-24-01734-s001.zip › ijms-2048174-supplementary.pdf]

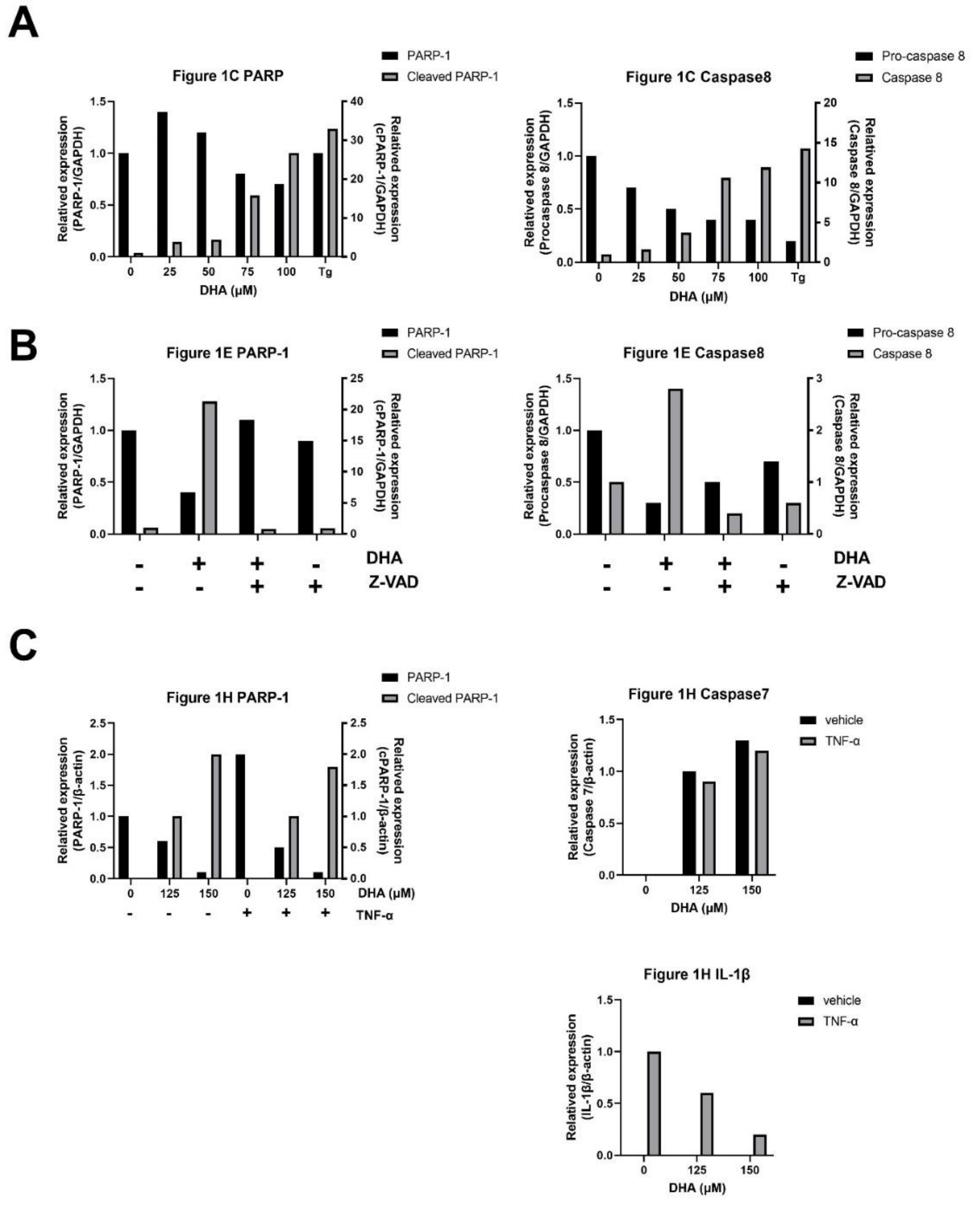

Figure S1. Graphs of quantified ratio values of each protein band shown in Figure 1. A ratio value was calculated by comparing the quantified protein band to the control (untreated). GAPDH (A, B) or β-actin (C) were used as controls, respectively.

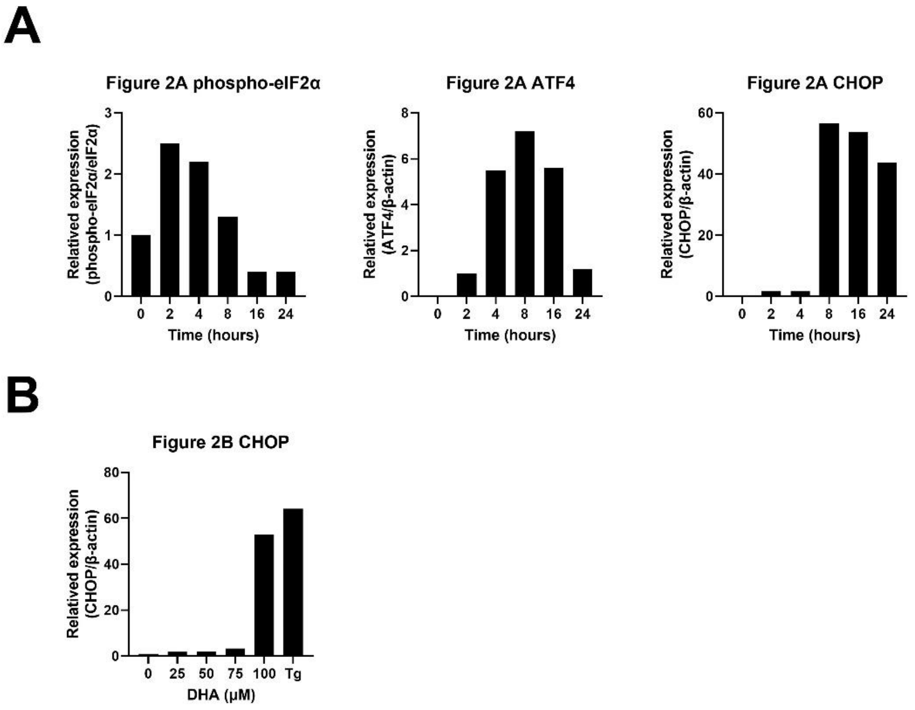

Figure S2. Graphs of quantified ratio values of each protein band shown in Figure 2. A ratio value was calculated by comparing the quantified protein band to the control (untreated). eIF2α or β-actin were used as controls, respectively.

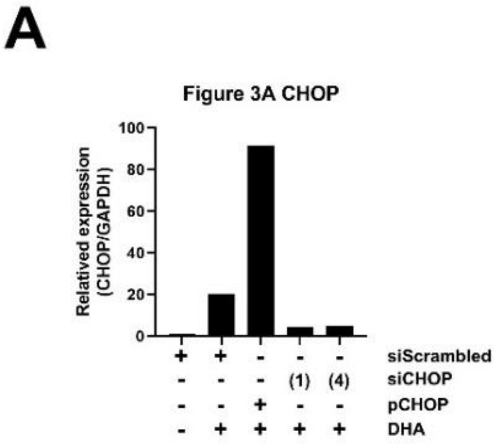

Figure S3. A graph of quantified ratio values of each protein band shown in Figure 3. A ratio value was calculated by comparing the quantified protein band to the control (untreated). GAPDH was used as a control.

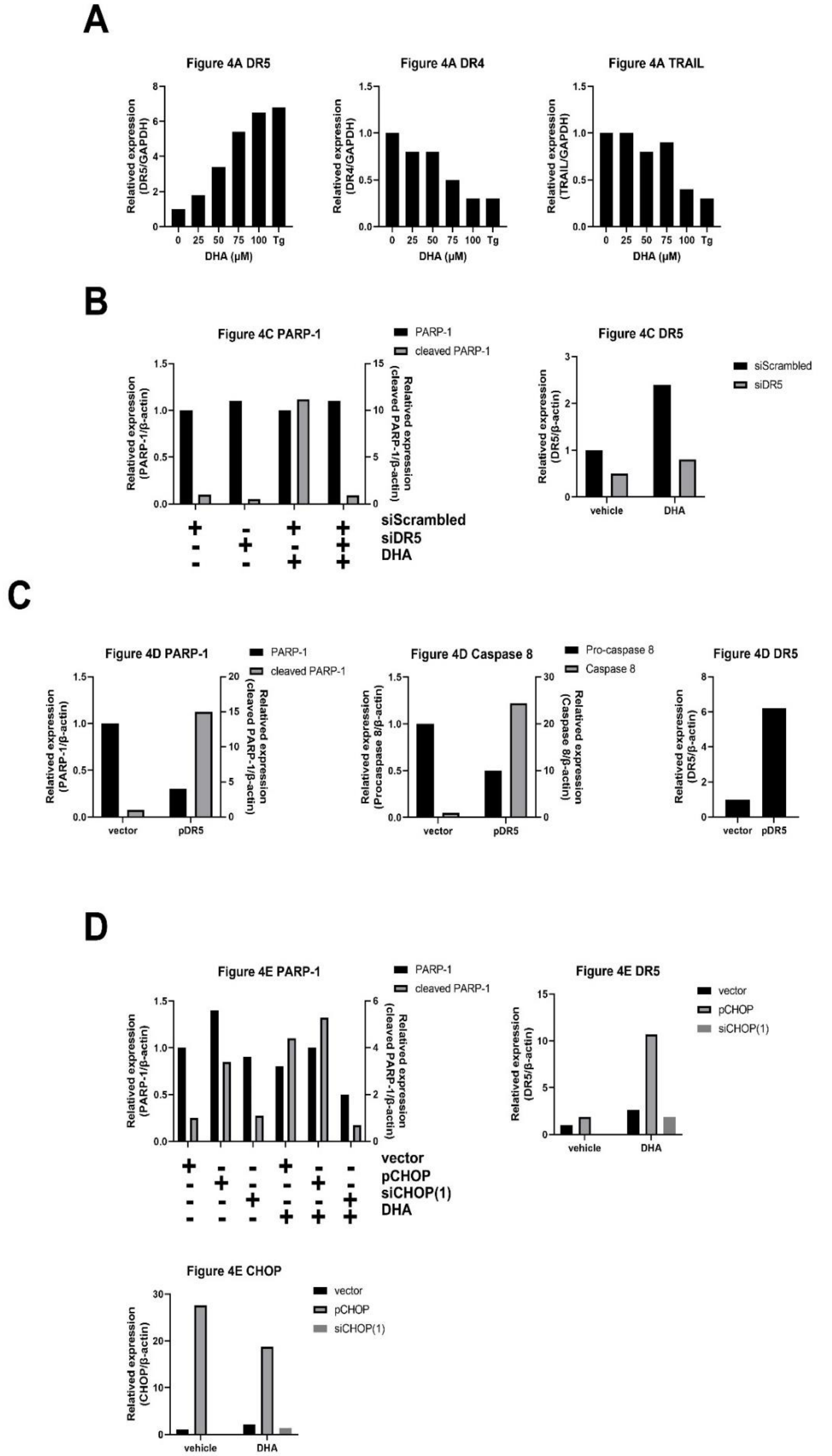

Figure S4. Graphs of quantified ratio values of each protein band shown in Figure 4. A ratio value was calculated by comparing the quantified protein band to the control (untreated). GAPDH (A) or β-actin (B, C, D) were used as controls, respectively.

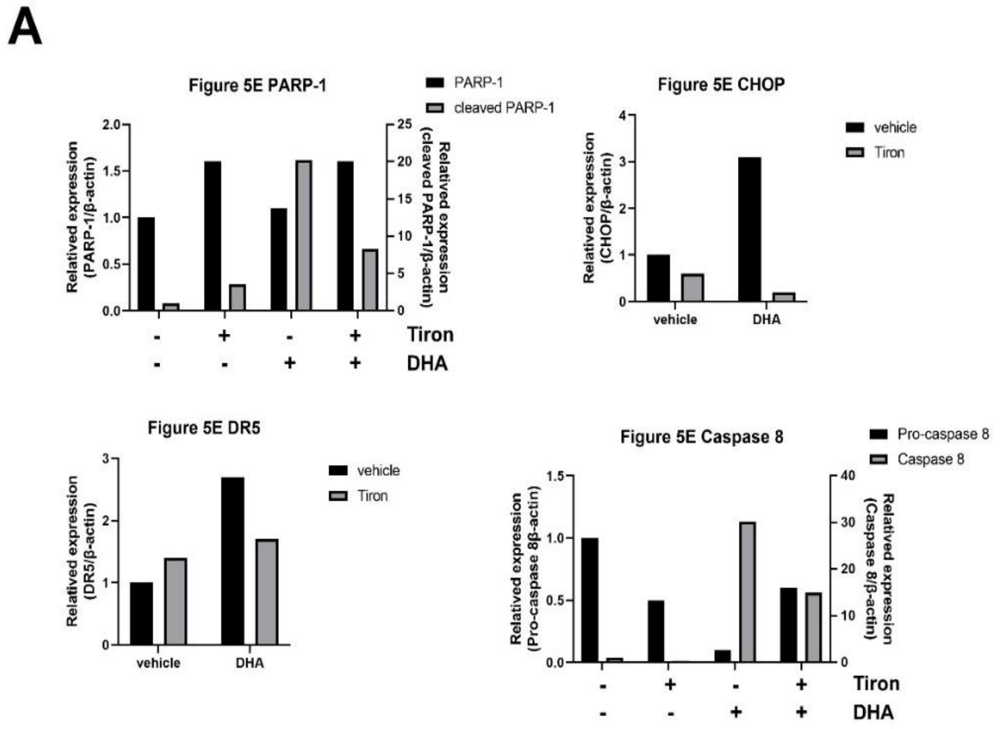

Figure S5. Graphs of quantified ratio values of each protein band shown in Figure 5. A ratio value was calculated by comparing the quantified protein band to the control (untreated). β-actin was used as a control.

A

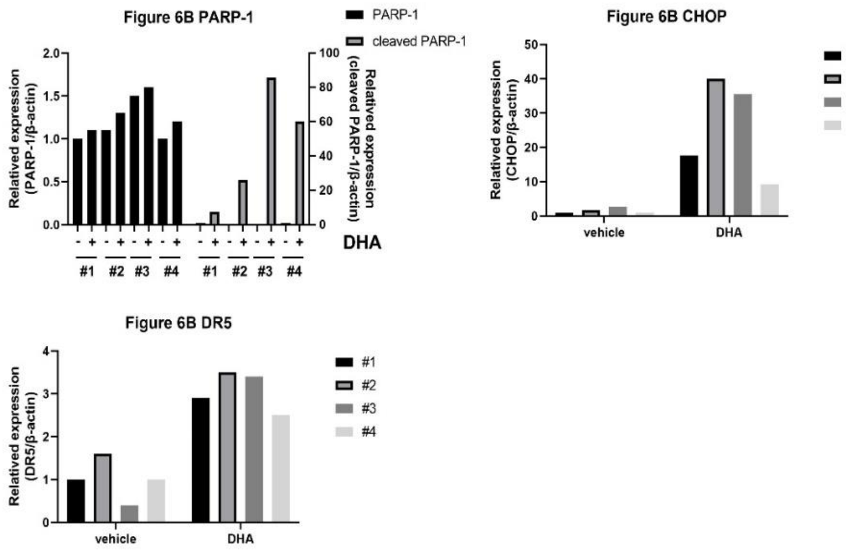

B

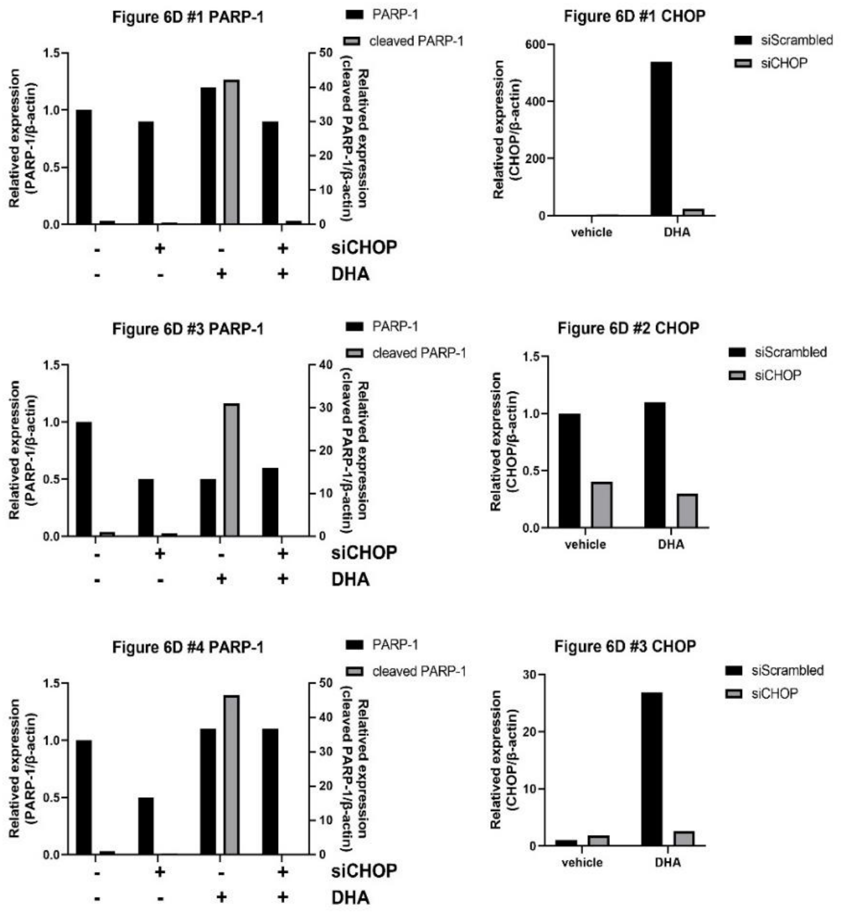

Figure S6. Graphs of quantified ratio values of each protein band shown in Figure 6. A ratio value was calculated by comparing the quantified protein band to the control (untreated). β-actin was used as a control.

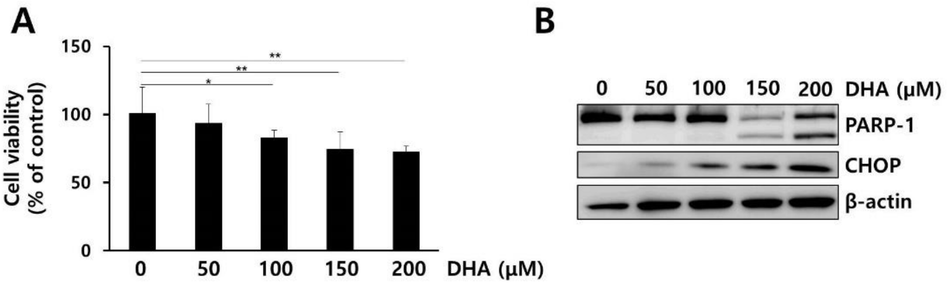

Figure S7. DHA induces apoptosis in A549 cells. (A) Cells were treated with various concentrations of DHA for 24 h and cell viability was determined by using Ez-cytox assay as described in the Materials and Methods. Data represent the means ± S.D. of three independent experiments performed in triplicate. A Significant difference is indicated as * $p<0.05$, ** $p<0.01$. (B) Cells were treated with various concentrations of DHA for 24 h and cell lysates were subjected to SDS-PAGE, followed by immunoblot analysis using antibodies specific to PARP-1, CHOP or β-actin.
